# Supplementary figures and images for: A novel sialic acid-binding adhesin present in multiple species contributes to the pathogenesis of Infective endocarditis
Source: PLoS Pathog. 2021 Jan 19;17(1):e1009222. doi: 10.1371/journal.ppat.1009222 (PMC7846122; doi:10.1371/journal.ppat.1009222)

**Uo5 secA2**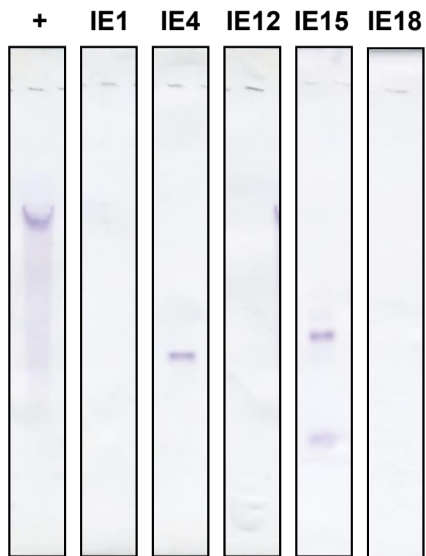**F0392 secA2**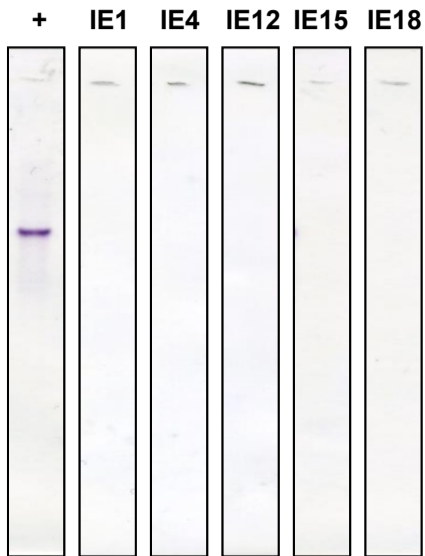**ATCC6249 secA2**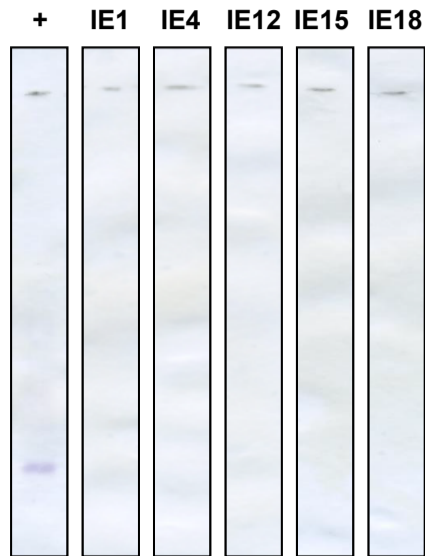

Supplement: S1 Fig — Southern blots of five different S. oralis subsp. oralis IE-isolates using three different DIG-labeled secA2 probes amplified from S. oralis Uo5, F0392 and ATCC6249, each of which represents one of the three distinct secA2 alleles identified within sequenced S. oralis strains. gDNA from the strains used to amplify each probe was used as a positive control for the appropriate blot (+). (PDF) [file ppat.1009222.s001.pdf]

A)

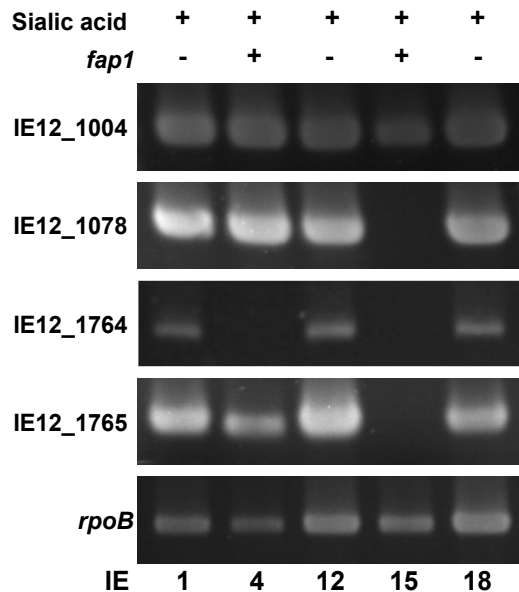

B)

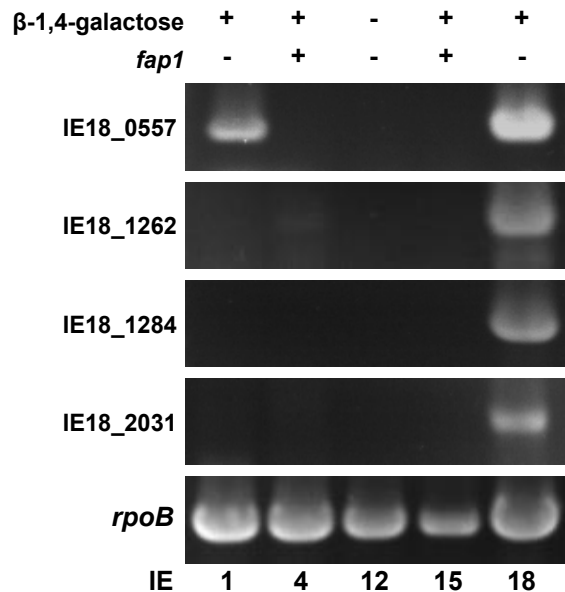

Supplement: S2 Fig — The presence of the four genes shared between IE12 and IE18 (A) and those unique to IE18 (B) in all five S. oralis subsp. oralis IE-isolates was determined by PCR. The distribution of these genes was correlated with the ability of the IE-isolates to bind sialic acid and β-1,4-linked galactose. Amplification of rpoB was used as a positive control. (PDF) [file ppat.1009222.s002.pdf]

**IE12**

**+**

**-**

**+**

**-**

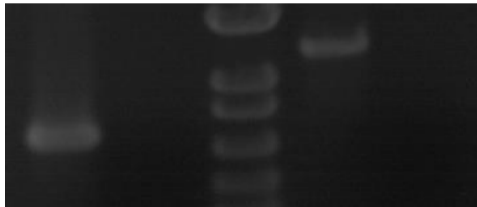

***rpoB***

**IE12\_1764**

**IE18**

**+**

**-**

**+**

**-**

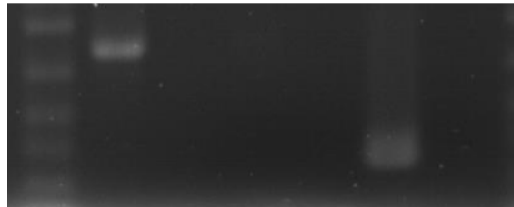

***rpoB***

**IE18\_0557**

Supplement: S3 Fig — Expression of IE12_1764 and IE18_0557 in IE12 and IE18, respectively, was analyzed by RT-PCR. In both cases, the expression of rpoB served as a positive control. To rule out DNA contamination, cDNA synthesis was performed in the absence of reverse transcriptase (-). (PDF) [file ppat.1009222.s003.pdf]

**IE18**

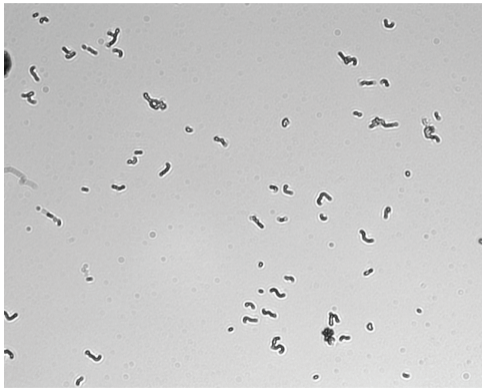

**IE18  $\Delta csh$ -like**

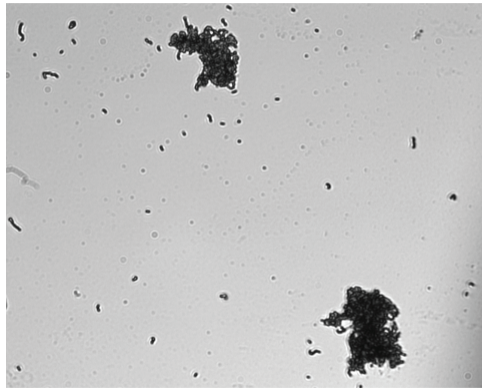

Supplement: S4 Fig — Gram-staining of IE18 and IE18 Δcsh-like shows bacterial aggregates that could not be disrupted by vortexing. Representative images captured using a Nikon eclipse Ti inverted microscope with a 40x objective. (PDF) [file ppat.1009222.s004.pdf]

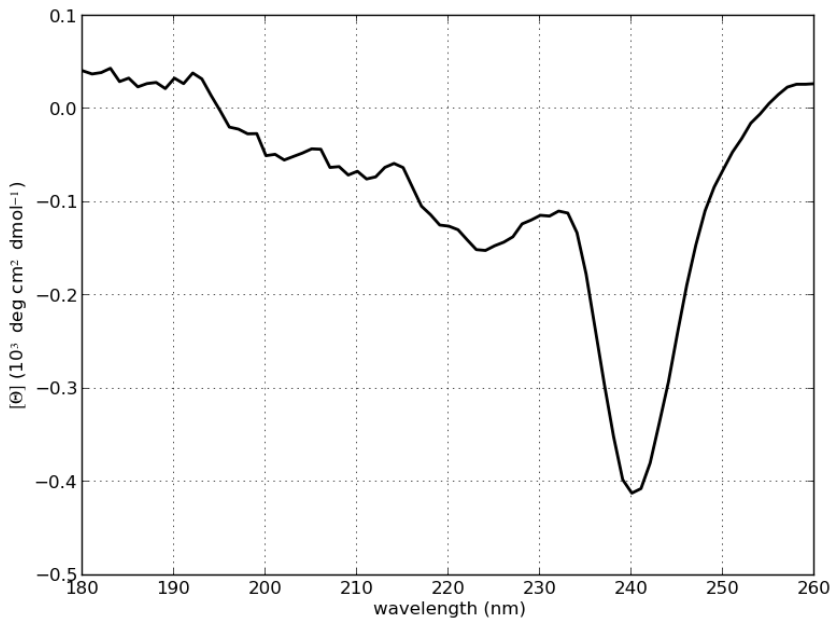

Supplement: S5 Fig — Representative CD spectra of the recombinantly expressed Csh_NRR at a concentration of 31.3 μM in PBS. (PDF) [file ppat.1009222.s005.pdf]

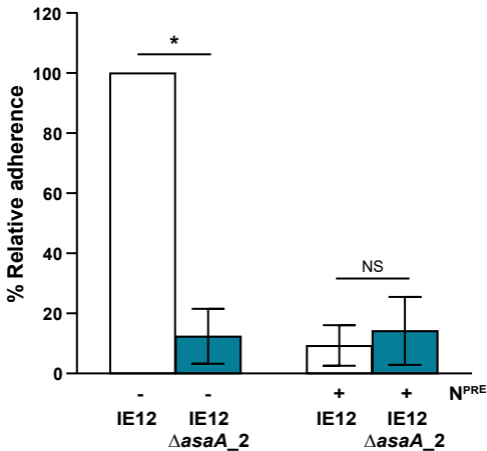

Supplement: S6 Fig — Adhesion of an independent IE12 asaA mutant to platelets pretreated with neuraminidase (NPRE) or PBS (-). Adherence is expressed as a percentage relative to binding of IE12 to untreated platelets. Values are the means for at least three independent experiments, each performed in triplicate, ± SD. Statistical significance was tested by two-tailed t Student’s t-test. *, P ≤ 0.0001. NS, not significant. (PDF) [file ppat.1009222.s006.pdf]

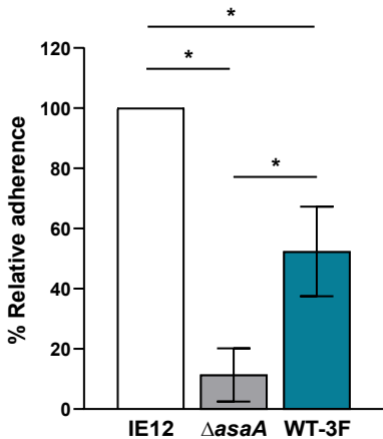

Supplement: S7 Fig — Adhesion to platelets of a strain expressing AsaA-3xFLAG (WT-3F) is reduced as compared to IE12. However, an asaA mutant is further reduced. Adherence is expressed as a percentage relative to binding of IE12. Values are the means for at least three independent experiments, each performed in triplicate, ± SD. Statistical significance was tested by two-tailed Student’s t-test. *, P ≤ 0.0007. (PDF) [file ppat.1009222.s007.pdf]

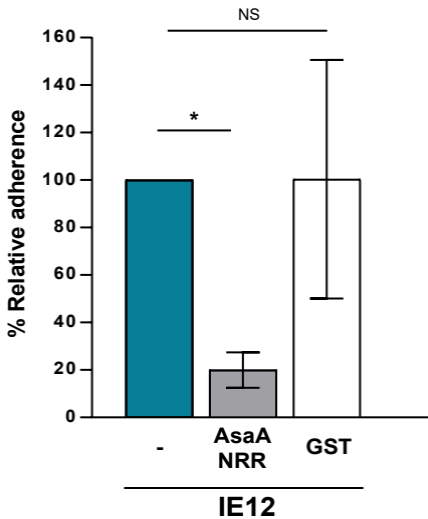

Supplement: S8 Fig — Unlike the addition of the recombinantly expressed So_AsaA_NRR (AsaA NRR,10 μM), the addition of 10 μM of GST alone did not significantly reduce binding of IE12 to platelets. Adherence is expressed as a percentage relative to binding of IE12 in the absence of So_AsaA_NRR or GST. Values are the means for at least three independent experiments, each performed in triplicate, ± SD. Statistical significance was tested by two-tailed Student’s t-test. *, P ≤ 0.0001; NS, not significant. (PDF) [file ppat.1009222.s008.pdf]

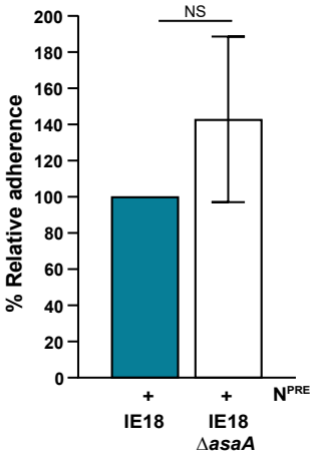

Supplement: S9 Fig — Adhesion of an IE18 asaA mutant to platelets pretreated with neuraminidase (NPRE). Adherence is expressed as a percentage relative to binding of IE18. Values are the means for at least three independent experiments, each performed in triplicate, ± SD. Statistical significance was tested by two-tailed t Student’s t-test. NS, not significant. (PDF) [file ppat.1009222.s009.pdf]
